# Secrecy Performance Enhancement for Underlay Cognitive Radio Networks Employing Cooperative Multi-Hop Transmission with and without Presence of Hardware Impairments

**DOI:** 10.3390/e21020217

**Published:** 2019-02-24

**Authors:** Phu Tran Tin, Dang The Hung, Tan N. Nguyen, Tran Trung Duy, Miroslav Voznak

**Affiliations:** 1 VSB—Technical University of Ostrava, 17. listopadu 15/2172, 708 33 Ostrava, Poruba, Czech Republic; 2 Faculty of Electronics Technology, Industrial University of Ho Chi Minh City, Ho Chi Minh City 71408, Vietnam; 3 Faculty of Radio-Electronics Engineering, Le Quy Don Technical University, Hanoi 11917, Vietnam; 4 Wireless Communications Research Group, Faculty of Electrical and Electronics Engineering, Ton Duc Thang University, Ho Chi Minh City 72912, Vietnam; 5 Department of Telecommunications, Posts and Telecommunications Institute of Technology, Ho Chi Minh City 71007, Vietnam

**Keywords:** physical-layer security, underlay cognitive radio, cooperative multi-hop transmission, secrecy outage probability, hardware impairments

## Abstract

In this paper, we consider a cooperative multi-hop secured transmission protocol to underlay cognitive radio networks. In the proposed protocol, a secondary source attempts to transmit its data to a secondary destination with the assistance of multiple secondary relays. In addition, there exists a secondary eavesdropper who tries to overhear the source data. Under a maximum interference level required by a primary user, the secondary source and relay nodes must adjust their transmit power. We first formulate effective signal-to-interference-plus-noise ratio (SINR) as well as secrecy capacity under the constraints of the maximum transmit power, the interference threshold and the hardware impairment level. Furthermore, when the hardware impairment level is relaxed, we derive exact and asymptotic expressions of end-to-end secrecy outage probability over Rayleigh fading channels by using the recursive method. The derived expressions were verified by simulations, in which the proposed scheme outperformed the conventional multi-hop direct transmission protocol.

## 1. Introduction

Security is one of the most important issues in wireless communication because of the broadcast nature of wireless medium. Conventionally, encryption/decryption algorithms that generate public/private keys are used to guarantee the security [1,2]. Recently, a security framework for the physical layer, called the wiretap channel or physical-layer security (PLS) [3,4,5,6,7,8,9,10,11], has been introduced as a potential solution. In PLS, difference between Shannon capacity of the data link and that of the eavesdropping link, named secrecy capacity, is commonly used to evaluate secrecy performance such as average secrecy capacity (ASC), secrecy outage probability (SOP) and probability of non-zero secrecy capacity (PNSC). Hence, to enhance the secrecy performance for wireless systems, researchers proposed efficient communication methods to increase channel capacity of the data links, and/or decrease that of the eavesdropping links. Indeed, in [12,13,14], opportunistic relay selection protocols are considered to enhance the quality of the data channels in one-hop and dual-hop relaying networks. In [15,16,17,18], the authors considered cooperative jamming approaches to reduce the data rate received at the eavesdroppers. The authors of [19,20,21,22,23,24,25] considered the secrecy performance enhancement for underlay cognitive radio (UCR) networks in which transmit power of secondary users (SUs) is limited by maximum interference levels required by primary users (PUs). The authors of [26,27,28,29] proposed secure communication protocols for two-way relay networks. In [30,31,32,33], the end-to-end secrecy performance of multi-hop relaying systems is investigated.

Thus far, most published works related to performance evaluation assume that transceiver hardware of wireless terminals is perfect. However, in practice, it suffers from impairments due to phase noises, amplifier–amplitude non-linearity and in phase and quadrature imbalance (IQI) [34,35,36], which significantly degrade the performance of wireless communication systems. In [37,38], the authors proposed various relay selection methods to compensate the impact of the hardware imperfection. The authors of [39] studied the outage performance of partial relay selection and opportunistic relay selection schemes in the UCR networks under the joint of hardware imperfection and interference constraint.

To the best of our knowledge, several published works evaluate the secrecy performance under the impact of imperfect transceiver hardware. In [40], the authors first studied the impact of the hardware imperfection on the secrecy capacity. In particular, the work in [40] considers the effects of IQI in one-hop OFDMA communication systems. The authors of [41] designed a secure massive MIMO system in the presence of a passive multiple-antenna eavesdropper and the hardware impairments. Reference [42] provided a power-efficient resource allocation algorithm for secure wireless-powered communication networks with the hardware noises. Taking hardware imperfection into account, the authors of [43] proposed an optimal power allocation strategy to maximize the instantaneous secrecy rate of a cooperative amplify-and-forward (AF) relaying scheme. In [44], we calculated PNSC of multi-hop relay networks over Nakagami-*m* fading channels in presence of the hardware impairments. The results in [44] show that the hardware impairments significantly affect on the PNSC performance.

However, there is no published work related to cooperative multi-hop PLS in the UCR networks. This motivated us to propose such a scheme and evaluate its performance. In the proposed protocol, named Cooperative Multi-Hop Transmission Protocol (CMT), a secondary source sends its data to a secondary destination via multiple secondary relays. In addition, in the secondary network, a secondary eavesdropper overhears the source data transmitted by the source and relay nodes. In addition, the secondary transmitters must adjust the transmit power to satisfy the interference constraint required by a PU and a maximal power threshold. The operation of the proposed scheme can be realized via one or many orthogonal time slots. At each time slot, the current transmitter finds an intended receiver that is nearest to the destination, and can receive the data securely and successfully. If this receiver is the destination, the data transmission ends. Otherwise, the procedure is repeated with the new selected transmitter. We also design a cooperative MAC method at each time slot for reversing the channel as well as selecting the potential receiver. For performance measurement, we first formulate the secrecy capacity under joint constraint of the limited interference and the hardware imperfection. When the hardware impairments are relaxed, we derive exact and asymptotic expressions of the end-to-end SOP over Rayleigh fading channels by using a recursive expression. Computer simulations were realized to verify the theoretical derivations as well as to show the advantages of the CMT method. The results show that the proposed scheme outperformed the conventional multi-hop direct transmission (MDT) protocol, and parameters such as the imperfect CSI estimations, the number of intermediate relays, the hardware impairment level and the position of the eavesdropper significantly affected the end-to-end SOP.

The rest of this paper is organized as follows. System model of the proposed scheme is described in Section 2. In Section 3, exact and asymptotic expressions of the end-to-end SOP for the MDT and CMT protocols are derived. The simulation results are presented in Section 4. Section 5 presents our conclusions.

## 2. System Model

**Figure 1:**
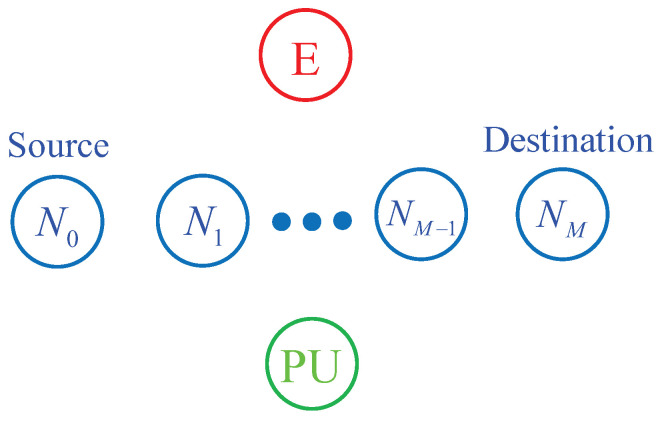
System model of the proposed protocol.

As illustrated in Figure 1, we consider an *M*-hop secondary network, where the source $\left(N_{0}\right)$ communicates with the destination $\left(N_{M}\right)$ via *M*− 1 relay nodes denoted by $N_{1}$, $N_{2}$, ..., $N_{M-1}$. The relay nodes are numbered according to their distances to the destination, i.e., the relay $N_{M-1}$ is nearest and the relay $N_{1}$ is the furthest. In UCR, the source and the relay nodes must adapt the transmit power so that the co-channel interference levels caused by their transmission are below a threshold $\left(I_{\mathrm{th}}\right)$ given by a primary user (PU). Moreover, the transmit power of the secondary transmitters is also limited by a maximum power $\left(P_{\mathrm{th}}\right)$. In addition, in the secondary network, the eavesdropper (E) attempts to overhear the source data transmitted by the secondary transmitters. Before describing the operation of the proposed protocol, we give assumptions used in this paper.

We assume that all of the relays are in the radio range of the source and destination nodes. We assume that all of the nodes have a single antenna, and the data transmission is hence split into orthogonal time slots. For ease of presentation and analysis, it is assumed that all of the nodes have the same structure, and the impairment levels are the same. We also assume that the eavesdropper is an active node, and hence the secondary nodes can estimate channel state information (CSI) between themselves and the node E [45]. Next, the data transmission between two secondary nodes is considered to be secure and successful if the obtained secrecy capacity is higher than a positive threshold $\left(R_{S}\right)$. Otherwise, the data are assumed to be intercepted, which is referred to as a secrecy outage event.

### 2.1. Channel and Hardware Impairment Models

Let $d_{N_{i},N_{j}}$, $d_{N_{i},\mathrm{PU}}$ and $d_{N_{i},E}$ denote distances of the $N_{i}\to N_{j}$, $N_{i}\to \mathrm{PU}$ and $N_{i}\to E$ links, respectively, where $i,j\in \left\{0,1,\ldots ,M-1,M\right\}$. We also denote $h_{N_{i},N_{j}}$, $h_{N_{i},\mathrm{PU}}$ and $h_{N_{i},E}$ as channel coefficients of $N_{i}\to N_{j}$, $N_{i}\to \mathrm{PU}$ and $N_{i}\to E$ links, respectively. Because the channels experience a Rayleigh fading distribution, the channel gains such as $\gamma_{i,j}=|h_{N_{i},N_{j}}{|}^{2}$, $\gamma_{i,P}=|h_{N_{i},\mathrm{PU}}{|}^{2}$ and $\gamma_{i,E}=|h_{N_{i},E}{|}^{2}$ follow exponential distributions. To take path-loss into account, we can model the parameters of the random variables (RVs) $\gamma_{i,j},\;\;\gamma_{i,P}$ and $\gamma_{i,E}$ as [46]: $\lambda_{i,j}=d_{N_{i},N_{j}}^{\beta }$, $\lambda_{i,P}=d_{N_{i},PU}^{\beta }$ and $\lambda_{i,E}=d_{N_{i},E}^{\beta }$, where β is path-loss exponent.

Considering the data transmission between the transmitter X and the receiver Y ($X\in \left\{N_{0},N_{1},\ldots ,N_{M-1}\right\}$, $Y\in \left\{N_{1},N_{2},\ldots ,N_{M},E,\mathrm{PU}\right\}$), the received data at Y is given as in [34,35,36]:

(1) $$\begin{array}{c} y=\sqrt{P_{X}}h_{X,Y}\left(x_{0}+\eta_{t,X}\right)+\eta_{r,Y}+\nu_{Y}, \end{array}$$

where $x_{0}$ is the source data, $P_{X}$ is the transmit power of X, $h_{X,Y}$ is channel coefficient of the X-Y link, $\eta_{t,X}$ and $\eta_{r,Y}$ are hardware noises at X and Y, respectively, and $\nu_{Y}$ is Gaussian noise at Y.

Similar to the work in [34,35,36], $\eta_{t,X}$, $\eta_{r,Y}$ and $\nu_{Y}$ are modeled as Gaussian random variables (RVs) with zero-mean and their variances are given, respectively, as

(2) $$\begin{array}{c} \mathrm{var}\left\{\eta_{t,X}\;\right\}\;=\;\tau_{t}^{2},\;\mathrm{var}\left\{\eta_{r,Y}\;\right\}\;=\;\tau_{r}^{2}P_{X}|h_{X,Y}{|}^{2},\mathrm{var}\left\{\nu_{Y}\;\right\}\;=\;\sigma_{0}^{2},\; \end{array}$$

where $\tau_{t}^{2}$ and $\tau_{r}^{2}$ are levels of the hardware impairments at X and Y, respectively.

From Equations (1) and (2), the instantaneous signal-to-interference-plus-noise ratio (SINR) is formulated by

(3) $$\begin{array}{cc} \Psi_{X,Y} & =\frac{P_{X}|h_{X,Y}{|}^{2}}{\left(\tau_{t}^{2}+\tau_{r}^{2}\right)P_{X}|h_{X,Y}{|}^{2}+\;\sigma_{0}^{2}}\\ & =\frac{P_{X}|h_{X,Y}{|}^{2}}{\kappa P_{X}|h_{X,Y}{|}^{2}+\;\sigma_{0}^{2}}, \end{array}$$

where $\kappa =\tau_{t}^{2}+\tau_{r}^{2}$ is the total hardware impairment level.

Let us consider the transmit power $P_{X}$ of the node X in the underlay CR network. Firstly, $P_{X}$ is below the maximum transmit power, i.e., $P_{X}\leq P_{\mathrm{th}}$. Secondly, the interference caused at the PU due to the transmission of the node X must be below the interference threshold $I_{\mathrm{th}}$, i.e.,

(4) $$\begin{array}{c} P_{X}\leq \frac{I_{\mathrm{th}}}{\left(1+\kappa \right)|h_{X,\mathrm{PU}}{|}^{2}}. \end{array}$$

Therefore, $P_{X}$ can be given as

(5) $$\begin{array}{cc} P_{X} & =\min\left(P_{\mathrm{th}},\frac{I_{\mathrm{th}}}{\left(1+\kappa \right)|h_{X,\mathrm{PU}}{|}^{2}}\right)\\ & =P_{\mathrm{th}}\min\left(1,\frac{\mu }{\left(1+\kappa \right)|h_{X,\mathrm{PU}}{|}^{2}}\right), \end{array}$$

where $\mu =I_{\mathrm{th}}/P_{\mathrm{th}}$ is assumed to be a constant.

Combining Equations (3) and (5) yields

(6) $$\begin{array}{c} \Psi_{X,Y}=\frac{P\min\left(1,\frac{\mu }{\left(1+\kappa \right)|h_{X,\mathrm{PU}}{|}^{2}}\right)|h_{X,Y}{|}^{2}}{\kappa P\min\left(1,\frac{\mu }{\left(1+\kappa \right)|h_{X,\mathrm{PU}}{|}^{2}}\right)|h_{X,Y}{|}^{2}+1}, \end{array}$$

where $P=P_{\mathrm{th}}/\sigma_{0}^{2}$.

From Equation (6), we can formulate the SINR for the $N_{i}\to N_{j}$ and $N_{i}\to E$ links, where $i,j\in \left\{0,1,\ldots ,M\right\}$, respectively, as

(7) $$\begin{array}{cc} \Psi_{i,j} & =\frac{P\min\left(1,\mu /\gamma_{i,P}\right)\gamma_{i,j}}{\kappa P\min\left(1,\mu /\gamma_{i,P}\right)\gamma_{i,j}+1},\\ \Psi_{i,E} & =\frac{P\min\left(1,\mu /\gamma_{i,P}\right)\gamma_{i,E}}{\kappa P\min\left(1,\mu /\gamma_{i,P}\right)\gamma_{i,E}+1}. \end{array}$$

Moreover, when the transceiver hardware of all the nodes is perfect, i.e., $\kappa =\kappa_{t}^{2}=\kappa_{r}^{2}=0$, we can rewrite Equation (7) as

(8) $$\begin{array}{cc} \Psi_{i,j} & =P\min\left(1,\frac{\mu }{\gamma_{i,P}}\right)\gamma_{i,j},\\ \Psi_{i,E} & =P\min\left(1,\frac{\mu }{\gamma_{i,P}}\right)\gamma_{i,E}. \end{array}$$

Hence, the secrecy capacity obtained at $N_{j}$ due to the transmission of $N_{i}$ is calculated as

(9) $$\begin{array}{cc} R_{i,j} & =\max\left(0,\log_{2}\left(1+\Psi_{i,j}\right)-\log_{2}\left(1+\Psi_{i,E}\right)\right)\\ & ={\left[\log_{2}\left(\frac{1+\Psi_{i,j}}{1+\Psi_{i,E}}\right)\right]}^{+}, \end{array}$$

where ${\left[x\right]}^{+}=\max\left(0,x\right)$.

From Equations (7) and (9), because $\Psi_{i,j}\overset{P\to +\infty }{\approx }1/\kappa$ and $\Psi_{i,E}\overset{P\to +\infty }{\approx }1/\kappa$, the secrecy capacity at high *P* regime can be given as

(10) $$\begin{array}{c} R_{i,j}\overset{P\to +\infty }{\approx }{\left[\log_{2}\left(\frac{1+1/\kappa }{1+1/\kappa }\right)\right]}^{+}=0. \end{array}$$

Moreover, as κ=0, we have

(11) $$\begin{array}{cc} R_{i,j} & ={\left[\log_{2}\left(\frac{1+P\min\left(1,\mu /\gamma_{i,P}\right)\gamma_{i,j}}{1+P\min\left(1,\mu /\gamma_{i,P}\right)\gamma_{i,E}}\right)\right]}^{+}\\ & \overset{P\to +\infty }{\approx }{\left[\log_{2}\left(\frac{\gamma_{i,j}}{\gamma_{i,E}}\right)\right]}^{+}. \end{array}$$

### 2.2. Operation of the Proposed Protocol

Next, we describe the operation of the proposed protocol, in which a MAC layer operation is designed to reverse the channel. Similar to the CoopMAC proposed in [47], in the first time slot, before transmitting the data, the source sends a request-to-send (RTS) message to the destination and all of the relays. By receiving this message, all of the nodes can estimate CSI between themselves and the source, calculate the instantaneous secrecy capacity by using Equation (9), and compare with $R_{S}$. It is assumed that the source can exactly estimate the channel coefficients of the interference and eavesdropping links, and include these values into the RTS message. If the destination can receive the source data securely and successfully, i.e., $R_{0,M}\geq R_{S}$, it will feedback a clear-to-send (CTS) message to inform. In this case, the source directly sends the data to the destination without using the relays. In the case where $R_{0,M}<R_{S}$, the destination has to generate a non-CTS message to request the help of the relays. Now, let us denote $U_{1}=\left\{N_{1_{1}},N_{1_{2}},\ldots ,N_{1_{r_{1}}}\right\}$ as set of the potential relays which can receive the data securely and successfully, i.e., $R_{0,1_{u}}\geq R_{S}$, where $u=1,2,\ldots ,r_{1}$, $0\leq r_{1}\leq M-1$, $N_{1_{u}}\in \left\{N_{1},N_{2},\ldots ,N_{M-1}\right\}$. To select the relay for the retransmission, we also propose a distributed relay selection method. Similar to the work in [48], the relay $N_{1_{u}}$ will set a timer given as

(12) $$\begin{array}{c} \omega_{1_{u}}=\frac{A}{\lambda_{1_{u},M}}, \end{array}$$

where A is a predetermined constant.

Then, the relay whose timer expires first will broadcast the CTS message, and it be selected to retransmit the data to the destination. We can observe from Equation (12) that the selected relay is nearest to the destination. It is worth noting that, if the set $U_{1}$ is empty ($r_{1}=0$), no relay node can retransmit the data to the destination, and this case is considereda secrecy outage event. In the case where $r_{1}\geq 1$, the operation will be repeated with the new source.

Generally, at the *k*th time slot $\left(k\geq 1\right)$, assume that the current source is $N_{i_{k}}$, $i_{k}\in \left\{0,1,\ldots ,M-1\right\}$ and $i_{1}=0$. Let $W_{k}=\left\{N_{i_{k}+1},N_{i_{k}+2},\ldots ,N_{M}\right\}$ denote set of relays from the node $N_{i_{k}+1}$ to the destination. Similarly, $N_{i_{k}}$ sends the RTS message to all of the nodes belonging to $W_{k}$. Then, if $R_{i_{k},M}\geq R_{S}$, the destination generates the CTS message, and $N_{i_{k}}$ will directly transmit the data to $N_{M}$. Otherwise, the potential relay which belongs to $W_{k}$ and is nearest to the destination will become the new source and repeat the process that $N_{i_{k}}$ did. Indeed, we denote $U_{k}$ as the set of the potential relays, i.e., $U_{k}=\left\{N_{k_{1}},N_{k_{2}},\ldots ,N_{k_{r_{k}}}\right\}$, where $U_{k}\subset W_{k}$, $0\leq r_{k}\leq M-i_{k}$. In addition, let us denote $Z_{k}=\left\{N_{k_{r_{k}+1}},N_{k_{r_{k}+2}},\ldots ,N_{M-i_{k}}\right\}$ as set of the nodes that cannot receive the data securely, where $k_{r_{k}+1}<k_{r_{k}+2}<\ldots <k_{M-i_{k}}$ and $N_{k_{M-i_{k}}}\equiv N_{M}$. Then, assume that $k_{1}<k_{2}<\ldots <k_{r_{k}}$ and $r_{k}\geq 1$, using the relay selection method described above, the relay $N_{k_{r}}$ will become the new source at the $\left(k+1\right)$th time slot.

This process is only stopped when $N_{M}$ can securely and successfully receive the data or there is no relay between the current source and the destination that can securely and successfully receive the data. It is noted that, to avoid the eavesdropper and combine the received data with maximal ratio combining (MRC) technique, the source and the selected relays use randomize-and-forward (RF) method [49,50].

In the proposed protocol, to select the successful relay at each time slot correctly, the CSI estimations over the data, interference and eavesdropping links are assumed to be perfect. However, in practice, the estimations may not be correct due to the time variation of the channel, finite number of pilot symbols and noises. Hence, we will discuss this problem in the next sub-section.

### 2.3. Imperfect Channel Estimation

In this subsection, we consider the imperfect channel estimation at the transmitter $N_{i}$ and the receiver $N_{j}$. From Equation (9), if $N_{j}$ wants to calculate the secrecy capacity $R_{i,j}$, it has to estimate the channel coefficient $h_{N_{i},N_{j}}$ correctly. In addition, $N_{i}$ has to estimate the channel coefficients $h_{N_{i},\mathrm{PU}}$ and $h_{N_{i},E}$, which are then sent to $N_{j}$ through the RTS message.

Let $h_{N_{i},N_{j}}^{e}$, $h_{N_{i},\mathrm{PU}}^{e}$ and $h_{N_{i},E}^{e}$ denote the estimated CSIs of $h_{N_{i},N_{j}}$, $h_{N_{i},\mathrm{PU}}^{e}$ and $h_{N_{i},E}$, respectively; the correlation between $h_{N_{i},N_{j}}^{e}$ and $h_{N_{i},N_{j}}$; $h_{N_{i},\mathrm{PU}}^{e}$ and $h_{N_{i},\mathrm{PU}}$; and $h_{N_{i},E}^{e}$ and $h_{N_{i},E}$ can be expressed, respectively as in [51]:

(13) $$\begin{array}{cc} h_{N_{i},N_{j}}^{e} & =\phi_{D}h_{N_{i},N_{j}}^{}+\sqrt{1-\phi_{D}^{2}}\varepsilon_{D},\\ h_{N_{i},\mathrm{PU}}^{e} & =\phi_{P}h_{N_{i},\mathrm{PU}}+\sqrt{1-\phi_{P}^{2}}\varepsilon_{P},\\ h_{N_{i},E}^{e} & =\phi_{E}h_{N_{i},E}+\sqrt{1-\phi_{E}^{2}}\varepsilon_{E}, \end{array}$$

where $\phi_{D}$, $\phi_{P}$ and $\phi_{E}$ are channel correlation factors, and $\varepsilon_{D}$, $\varepsilon_{P}$ and $\varepsilon_{E}$ are estimation errors. We can observe that if $\phi_{D}=\phi_{P}=\phi_{E}=1$, all of the channel estimations are perfect. If $\phi_{D}<1,\phi_{P}<1,\phi_{E}<1$, the channel estimations have errors, and the estimated secrecy capacity in Equation (9) is written by

(14) $$\begin{array}{c} R_{i,j}^{e}={\left[\log_{2}\left(\frac{1+P\min\left(1,\frac{\mu }{\gamma_{i,P}^{e}}\right)\gamma_{i,j}^{e}}{1+P\min\left(1,\frac{\mu }{\gamma_{i,P}^{e}}\right)\gamma_{i,E}^{e}}\right)\right]}^{+}, \end{array}$$

where $\gamma_{i,j}^{e}=|h_{N_{i},N_{j}}^{e}{|}^{2}$, $\gamma_{i,P}^{e}=|h_{N_{i},\mathrm{PU}}^{e}{|}^{2}$ and $\gamma_{i,E}^{e}=|h_{N_{i},E}^{e}{|}^{2}$. Again, we note that the CSI estimation errors may lead to the incorrect relay selection, which would degrade the system performance.

### 2.4. Multi-Hop Direct Transmission Protocol

To show the advantages of the proposed protocol, we compared the secrecy performance of the proposed protocol with that of the conventional multi-hop direct transmission protocol (MDT) [44]. In the MDT scheme, the data are transmitted hop-by-hop from the source to the destination. Particularly, the data transmission is split into *M* orthogonal time slots. At the *m*th time slot, where m=1,2,…,M, the node $N_{m}$ transmits the source data to the node $N_{m+1}$. If the communication between $N_{m}$ and $N_{m+1}$ is secure and successful, $N_{m+1}$ will forward the data to the next hop in the next time slot. Otherwise, the data transmission is insecure and the secrecy outage event occurs. Similar to the MCT protocol, the source and relays in the MDT protocol use the RF technique.

## 3. Performance Analysis

Firstly, we can formulate SOP of the $N_{i}\to N_{j}$ link as

(15) $$\begin{array}{cc} {\mathrm{SOP}}_{i,j}^{\mathrm{DT}} & =\mathrm{Pr}\left(R_{i,j}<R_{S}\right)\\ & =\mathrm{Pr}\left(\frac{1+\Psi_{i,j}}{1+\Psi_{i,E}}<\rho \right), \end{array}$$

where $\rho =2^{R_{S}}\left(\rho >1\right)$.

From Equations (9) and (15), it is straightforward that, if κ>0, then

(16) $$\begin{array}{c} {\mathrm{SOP}}_{i,j}^{\mathrm{DT}}\overset{P\to +\infty }{\approx }1. \end{array}$$

When the transceiver hardware is perfect $\left(\kappa =0\right)$, we can derive the exact closed-form expression for ${\mathrm{SOP}}_{i,j}^{\mathrm{DT}}$. At first, setting $x=\gamma_{i,P}$, ${\mathrm{SOP}}_{i,j}^{\mathrm{DT}}$ conditioned on *x* can be given by

(17) $$\begin{array}{c} {\mathrm{SOP}}_{i,j}^{\mathrm{DT}}\left(x\right)=\mathrm{Pr}\left(\gamma_{i,j}<\frac{\rho -1}{P\min\left(1,\mu /x\right)}+\rho \gamma_{i,E}\right). \end{array}$$

Due to the independence of $\gamma_{i,j}$ and $\gamma_{i,E}$, we can write

(18) $$\begin{array}{cc} & {\mathrm{SOP}}_{i,j}^{\mathrm{DT}}\left(x\right)=\int_{0}^{+\infty }f_{\gamma_{i,E}}\left(y\right)F_{\gamma_{i_{j}}}\left(\frac{\rho -1}{P\min\left(1,\mu /x\right)}+\rho y\right)dy. \end{array}$$

Substituting probability density function (PDF) of the exponential RV $\gamma_{i,E}$$\left(f_{\gamma_{i,E}}\left(y\right)=\lambda_{i,E}\exp\left(-\lambda_{i,E}y\right)\right)$, and the cumulative distribution function (CDF) of the exponential RV $\gamma_{i,j}$$\gamma_{i,E}$$\left(F_{\gamma_{i,j}}\left(y\right)=1-\exp\left(-\lambda_{i,j}y\right)\right)$ into Equation (18), after some manipulations, we obtain

(19) $$\begin{array}{c} \;{\mathrm{SOP}}_{i,j}^{\mathrm{DT}}\left(x\right)=1-\frac{\lambda_{i,E}}{\lambda_{i,E}+\lambda_{i,j}\rho }\exp\left(\;-\frac{\rho -1}{P\min\left(1,\mu /x\right)}\;\right).\; \end{array}$$

Then, ${\mathrm{SOP}}_{i,j}^{\mathrm{DT}}$ can be obtained from ${\mathrm{SOP}}_{i,j}^{\mathrm{DT}}\left(x\right)$ by

(20) $$\begin{array}{c} {\mathrm{SOP}}_{i,j}^{\mathrm{DT}}=\int_{0}^{+\infty }{\mathrm{SOP}}_{i,j}^{\mathrm{DT}}\left(x\right)f_{\gamma_{i,P}}\left(x\right)dx. \end{array}$$

Substituting Equation (19) and $f_{\gamma_{i,P}}\left(y\right)=\lambda_{i,P}\exp\left(-\lambda_{i,P}y\right)$ into Equation (20), we obtain an exact closed-form expression of ${\mathrm{SOP}}_{i,j}^{\mathrm{DT}}$ as

(21) $$\begin{array}{c} {\mathrm{SOP}}_{i,j}^{\mathrm{DT}}=\;\int_{0}^{\mu }\left(1-\frac{\lambda_{i,E}}{\lambda_{i,E}+\lambda_{i,j}\rho }\exp\left(-\frac{\rho -1}{P}\right)\right)\lambda_{i,P}\exp\left(-\lambda_{i,P}x\right)dx\\ +\int_{\mu }^{+\infty }\left(1-\frac{\lambda_{i,E}}{\lambda_{i,E}+\lambda_{i,j}\rho }\exp\left(-\frac{\rho -1}{P\mu }x\right)\right)\lambda_{i,P}\exp\left(-\lambda_{i,P}x\right)dx\\ \;=\;1\;-\;\frac{\lambda_{i,E}}{\lambda_{i,E}\;+\;\lambda_{i,j}\rho }\;\;\left[\;\left(1-\exp\;\left(\;-\lambda_{i,P}\mu \right)\;\right)\exp\;\;\left(\;\;-\lambda_{i,j}\frac{\rho \;-\;1}{P}\;\right)\;+\;\frac{\lambda_{i,P}P\mu }{\lambda_{i,P}P\mu \;+\;\lambda_{i,j}\left(\rho \;-\;1\right)}\exp\;\;\left(\;\;-\lambda_{i,P}\mu -\lambda_{i,j}\frac{\rho \;-\;1}{P}\;\;\right)\;\right]\;\;.\; \end{array}$$

Furthermore, using the approximation in Equation (11), an asymptotic closed-form expression for ${\mathrm{SOP}}_{i,j}^{\mathrm{DT}}$ at high *P* values can be provided by

(22) $$\begin{array}{c} {\mathrm{SOP}}_{i,j}^{\mathrm{DT}}\overset{P\to +\infty }{\approx }\mathrm{Pr}\left(\frac{\gamma_{i,j}}{\gamma_{i,E}}<\rho \right)=1-\frac{\lambda_{i,E}}{\lambda_{i,E}+\lambda_{i,j}\rho }. \end{array}$$

### 3.1. Multi-hop Direct Transmission Protocol (MDT)

Because the transmission on each hop is independent, the end-to-end SOP of the MDT protocol can be given as

(23) $$\begin{array}{c} {\mathrm{SOP}}_{0,M}^{\mathrm{MDT}}=1-\prod_{m=1}^{M}\left(1-{\mathrm{SOP}}_{m-1,m}^{\mathrm{DT}}\right). \end{array}$$

As κ=0, substituting Equation (21) into Equation (23), we obtain an exact closed-form expression for the end-to-end SOP of the MDT protocol as

(24) $$\begin{array}{c} {\mathrm{SOP}}_{0,M}^{\mathrm{MDT}}\;=\;1\;-\;\prod_{m=1}^{M}\;\left\{\frac{\lambda_{m-1,E}}{\lambda_{m-1,E}+\lambda_{i,j}\rho }\;\left[\begin{array}{c} \left(1-\exp\left(-\lambda_{m-1,P}\mu \right)\right)\exp\left(-\lambda_{m-1,m}\frac{\rho -1}{P}\right)\\ +\frac{\lambda_{m-1,P}P\mu }{\lambda_{m-1,P}P\mu +\lambda_{m-1,m}\left(\rho -1\right)}\exp\left(-\lambda_{m-1,P}\mu -\lambda_{m-1,m}\frac{\rho -1}{P}\right) \end{array}\right]\right\}. \end{array}$$

At high *P* regions, using Equation (22), an approximate expression for Equation (24) can be obtained by

(25) $$\begin{array}{c} {\mathrm{SOP}}_{0,M}^{\mathrm{MDT}}\overset{P\to +\infty }{\approx }1-\prod_{m=1}^{M}\frac{\lambda_{m-1,E}}{\lambda_{m-1,E}+\lambda_{m-1,m}\rho }. \end{array}$$

### 3.2. Cooperative Multi-Hop Transmission Protocol (CMT)

In the CMT protocol, the end-to-end SOP is expressed by a recursive expression as follows:

(26) $$\begin{array}{cc} {\mathrm{SOP}}_{N_{i_{k}},U_{k}}^{\mathrm{CMT}} & =\sum_{U_{k}}^{}\mathrm{Pr}\left(\begin{array}{c} \frac{1+\Psi_{i_{k},k_{1}}}{1+\Psi_{i_{k},E}}\geq \rho ,\frac{1+\Psi_{i_{k},k_{2}}}{1+\Psi_{i_{k},E}}\geq \rho ,\ldots ,\frac{1+\Psi_{i_{k},k_{r_{k}}}}{1+\Psi_{i_{k},E}}\geq \rho ,\\ \frac{1+\Psi_{i_{k},k_{r_{k}+1}}}{1+\Psi_{i_{k},E}}<\rho ,\frac{1+\Psi_{i_{k},k_{r_{k}+2}}}{1+\Psi_{i_{k},E}}<\rho ,\ldots ,\frac{1+\Psi_{i_{k},k_{M-i_{k}}}}{1+\Psi_{i_{k},E}}<\rho \end{array}\right)\\ & =\sum_{U_{k}}^{}\mathrm{Pr}\left(\begin{array}{c} \frac{1+P\min\left(1,\mu /\gamma_{i_{k},P}\right)\gamma_{i_{k},k_{1}}}{1+P\min\left(1,\mu /\gamma_{i_{k},P}\right)\gamma_{i_{k},E}}\geq \rho ,\frac{1+P\min\left(1,\mu /\gamma_{i_{k},P}\right)\gamma_{i_{k},k_{2}}}{1+P\min\left(1,\mu /\gamma_{i_{k},P}\right)\gamma_{i_{k},E}}\geq \rho ,\ldots ,\\ \frac{1+P\min\left(1,\mu /\gamma_{i_{k},P}\right)\gamma_{i_{k},k_{r_{k}}}}{1+P\min\left(1,\mu /\gamma_{i_{k},P}\right)\gamma_{i_{k},E}}\geq \rho ,\\ \frac{1+P\min\left(1,\mu /\gamma_{i_{k},P}\right)\gamma_{i_{k},k_{r_{k}+1}}}{1+P\min\left(1,\mu /\gamma_{i_{k},P}\right)\gamma_{i_{k},E}}<\rho ,\frac{1+P\min\left(1,\mu /\gamma_{i_{k},P}\right)\gamma_{i_{k},k_{r_{k}+2}}}{1+P\min\left(1,\mu /\gamma_{i_{k},P}\right)\gamma_{i_{k},E}}<\rho ,\ldots ,\\ \frac{1+P\min\left(1,\mu /\gamma_{i_{k},P}\right)\gamma_{i_{k},k_{M-i_{k}}}}{1+P\min\left(1,\mu /\gamma_{i_{k},P}\right)\gamma_{i_{k},E}}<\rho \end{array}\right), \end{array}$$

where ${\mathrm{SOP}}_{N_{i_{k}},U_{k}}^{\mathrm{CMT}}$ is SOP at *k*th time slot, k=1,2,…,M. Then, the end-to-end SOP of the CMT protocol is given as

(27) $$\begin{array}{c} {\mathrm{SOP}}_{0,M}^{\mathrm{CMT}}={\mathrm{SOP}}_{N_{0},U_{1}}^{\mathrm{CMT}}. \end{array}$$

Before calculating ${\mathrm{SOP}}_{N_{i_{k}},U_{k}}^{\mathrm{CMT}}$, we give an example with M=3, where ${\mathrm{SOP}}_{0,3}^{\mathrm{CMT}}$ is expressed by

(28) $$\begin{array}{cc} {\mathrm{SOP}}_{0,3}^{\mathrm{CMT}} & ={\mathrm{SOP}}_{N_{0},\left\{\emptyset \right\}}^{\mathrm{CMT}}+{\mathrm{SOP}}_{N_{0},\left\{N_{1}\right\}}^{\mathrm{CMT}}+{\mathrm{SOP}}_{N_{0},\left\{N_{2}\right\}}^{\mathrm{CMT}}\\ & +{\mathrm{SOP}}_{N_{0},\left\{N_{1},N_{2}\right\}}^{\mathrm{CMT}}. \end{array}$$

Equation (28) shows that there are 04 possible cases for the set $U_{1}$, i.e., $U_{1}=\left\{\emptyset \right\}$, $U_{1}=\left\{N_{1}\right\}$, $U_{1}=\left\{N_{2}\right\}$, $U_{1}=\left\{N_{1},N_{2}\right\}$. In Equation (28), the terms ${\mathrm{SOP}}_{N_{0},\left\{\emptyset \right\}}^{\mathrm{CMT}}$ and ${\mathrm{SOP}}_{N_{0},\left\{N_{2}\right\}}^{\mathrm{CMT}}$ can be calculated as in (32). Considering the term ${\mathrm{SOP}}_{N_{0},\left\{N_{1}\right\}}^{\mathrm{CMT}}$, which can be written by

(29) $$\begin{array}{cc} {\mathrm{SOP}}_{N_{0},\left\{N_{1}\right\}}^{\mathrm{CMT}} & ={\mathrm{SOP}}_{N_{1},U_{2}}^{\mathrm{CMT}}={\mathrm{SOP}}_{N_{1},\left\{\emptyset \right\}}^{\mathrm{CMT}}+{\mathrm{SOP}}_{N_{1},\left\{N_{2}\right\}}^{\mathrm{CMT}}. \end{array}$$

In Equation (29), there are two possible cases for the set $U_{2}$, i.e., $U_{2}=\left\{\emptyset \right\}$, $U_{2}=\left\{N_{2}\right\}$, and ${\mathrm{SOP}}_{N_{1},\left\{\emptyset \right\}}^{\mathrm{CMT}}$ and ${\mathrm{SOP}}_{N_{1},\left\{N_{2}\right\}}^{\mathrm{CMT}}$ are SOP at the second time slots. In addition, ${\mathrm{SOP}}_{N_{1},\left\{\emptyset \right\}}^{\mathrm{CMT}}$ is calculated by Equation (32), while ${\mathrm{SOP}}_{N_{1},\left\{N_{2}\right\}}^{\mathrm{CMT}}$ is expressed by

(30) $$\begin{array}{c} {\mathrm{SOP}}_{N_{1},\left\{N_{2}\right\}}^{\mathrm{CMT}}={\mathrm{SOP}}_{2,3}^{\mathrm{DT}}, \end{array}$$

where, because the transmission between $N_{2}$ and $N_{3}$ is direct, Equation (21) is used to calculate ${\mathrm{SOP}}_{N_{1},\left\{N_{2}\right\}}^{\mathrm{CMT}}$.

Next, let us consider the term ${\mathrm{SOP}}_{N_{0},\left\{N_{1},N_{2}\right\}}^{\mathrm{CMT}}$ in Equation (28), where the relay $N_{2}$ will be selected for retransmitting the data to the destination. Similar to Equation (30), we have

(31) $$\begin{array}{c} {\mathrm{SOP}}_{N_{0},\left\{N_{1},N_{2}\right\}}^{\mathrm{CMT}}={\mathrm{SOP}}_{2,3}^{\mathrm{DT}}. \end{array}$$

Now, the recursive expression of ${\mathrm{SOP}}_{N_{i_{k}},U_{k}}^{\mathrm{CMT}}$ is given as in Lemma 1.

**Lemma** **1.** *When κ=0, ${\mathrm{SOP}}_{N_{i_{k}},U_{k}}^{\mathrm{CMT}}$ can be expressed as*

(32) SOPNik,UkCMT=∑Ukλik,Eλik,E+∑t=1rkλik,ktρexp−∑t=1rkλik,ktρ−1P1−exp−λik,Pμ+λik,PPμλik,PPμ+∑t=1rkλik,ktρ−1exp−λik,Pμ−∑t=1rkλik,ktρ−1P+∑Uk∑v=1M−ik−rk−1v∑Nj1,…,Njv∈Zkj1<j2<…<jvM−ik−rkλik,Eλik,E+∑t=1vλik,jv+∑t=1rkλik,ktρ×exp−∑t=1vλik,jv+∑t=1rkλik,ktρ−1P1−exp−λik,Pμ+λik,PPμλik,PPμ+λik,E+∑t=1rkλik,ktρ−1exp−λik,Pμ−∑t=1vλik,jv+∑t=1rkλik,ktρ−1P.

**Proof.**  At first, we set $x=\gamma_{i_{k},E}$ and $y=\gamma_{i_{k},P}$, and ${\mathrm{SOP}}_{N_{i_{k}},U_{k}}^{\mathrm{CMT}}$ conditioned on *x* and *y* can be given by

(33) SOPNik,UkCMTx,y=∑Uk∏t=1rkexp−λik,ktρ−1Pmin1,μ/y+ρx∏v=1M−ik−rk1−exp−λik,kvρ−1Pmin1,μ/y+ρx=∑Ukexp−∑t=1rkλik,ktρ−1Pmin1,μ/y+ρx+∑Uk∑v=1M−ik−rk−1v∑Nj1,…,Njv∈Zkj1<j2<…<jvM−ik−rkexp−∑t=1vλik,jv+∑t=1rkλik,ktρ−1Pmin1,μ/y+ρx.

Then, ${\mathrm{SOP}}_{N_{i_{k}},U_{k}}^{\mathrm{CMT}}$ is obtained from ${\mathrm{SOP}}_{N_{i_{k}},U_{k}}^{\mathrm{CMT}}\left(x,y\right)$ by

(34) $$\begin{array}{c} {\mathrm{SOP}}_{N_{i_{k}},U_{k}}^{\mathrm{CMT}}=\int_{0}^{+\infty }f_{\gamma_{i_{k},P}}\left(y\right)\underset{I_{1}}{\underset{︸}{\left[\int_{0}^{+\infty }f_{\gamma_{i_{k},E}}\left(x\right){\mathrm{SOP}}_{N_{i_{k}},U_{k}}^{\mathrm{CMT}}\left(x,y\right)dx\right]}}dy. \end{array}$$

Let us consider the integral $I_{1}$ marked in Equation (34); combining the PDF $f_{\gamma_{i_{k},E}}$ and Equation (33), after some careful manipulations, we obtain

(35) I1=∑Ukλik,Eλik,E+∑t=1rkλik,ktρexp−∑t=1rkλik,ktρ−1Pmin1,μ/y+∑Uk∑v=1M−ik−rk−1v∑Nj1,…,Njv∈Zkj1<j2<…<jvM−ik−rkλik,Eλik,E+∑t=1vλik,jv+∑t=1rkλik,ktρ×exp−∑t=1vλik,jv+∑t=1rkλik,ktρ−1Pmin1,μ/y.

Next, substituting Equation (35) into Equation (34), and after some manipulations, we obtain Equation (32) and finish the proof.

Then, at high transmit power, i.e., P→+∞, using Equation (11), and with the same manner as derived in Equation (32), an asymptotic expression of ${\mathrm{SOP}}_{N_{i_{k}},U_{k}}^{\mathrm{CMT}}$ can be given by

(36) SOPNik,UkCMT≈P→+∞∑Ukλik,Eλik,E+∑t=1rkλik,ktρ+∑Uk∑v=1M−ik−rk−1v∑Nj1,…,Njv∈Zkj1<j2<…<jvM−ik−rkλik,Eλik,E+∑t=1vλik,jv+∑t=1rkλik,ktρ.

Finally, it is worth noting from Equations (25) and (36) that the asymptotic formulas of SOP do not depend on *P*. □

## 4. Simulation Results

In this section, we present various Monte Carlo simulations to verify the theoretical results derived in Section 3. For the simulation environment, we considered a two-dimensional network in which the co-ordinate of the node $N_{i}\left(i=0,1,\ldots ,M\right)$, the primary user, and the eavesdropper are $\left(0,i/M\right)$, $\left(x_{\mathrm{PU}},y_{\mathrm{PU}}\right)$ and $\left(x_{E},y_{E}\right)$, respectively. To focus on investigating the impact of the important system parameters on the system performance, in all of the simulations, the path-loss exponent β was fixed by 3.

**Figure 2:**
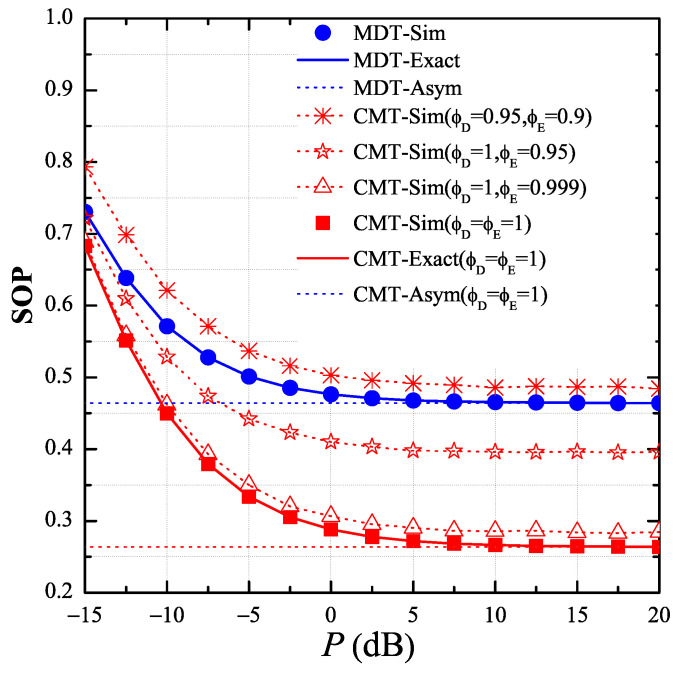
End-to-end secrecy outage probability (SOP) as function of *P* in dB when $P\in \left[-15\;\mathrm{dB},\;20\;\mathrm{dB}\right]$, μ=0.5, M=3, $R_{S}=0.2$, κ=0, $\left(x_{\mathrm{PU}},y_{\mathrm{PU}}\right)=\left(-0.5,\;-1\right)$ and $\left(x_{E},y_{E}\right)=\left(0.5,\;0.5\right)$.

In Figure 2, we present the end-to-end SOP of the MDT and CMT protocols as a function of the transmit SNR $\left(P=P_{\mathrm{th}}/\sigma_{0}^{2}\right)$ in dB, and investigate the impact of the CSI estimation errors on the secrecy performance. In this simulation, we assumed the CSI estimations of the interference links are correct, i.e., $\phi_{P}=1$, and the transceiver hardware is perfect, i.e., κ=0. We also set the simulation parameters as follows: the target rate $R_{S}=0.2$, the ratio μ=0.5, and the number of hops *M* = 3. In addition, we placed the primary user and the eavesdropper at the positions $\left(-0.5,\;-1\right)$ and $\left(0.5,\;0.5\right)$, respectively. As shown in Figure 2, when the estimations of the data and eavesdropping channels were correct, i.e., $\phi_{D}=\phi_{E}=1$, the performance of the proposed protocol (CMT) was much better than that of the MDT protocol. However, the SOP performance of the CMT protocol significantly decreased with the CSI estimation errors. Moreover, when $\phi_{D}=0.95$ and $\phi_{E}=0.9$, the MDT protocol outperformed the proposed protocol.

**Figure 3:**
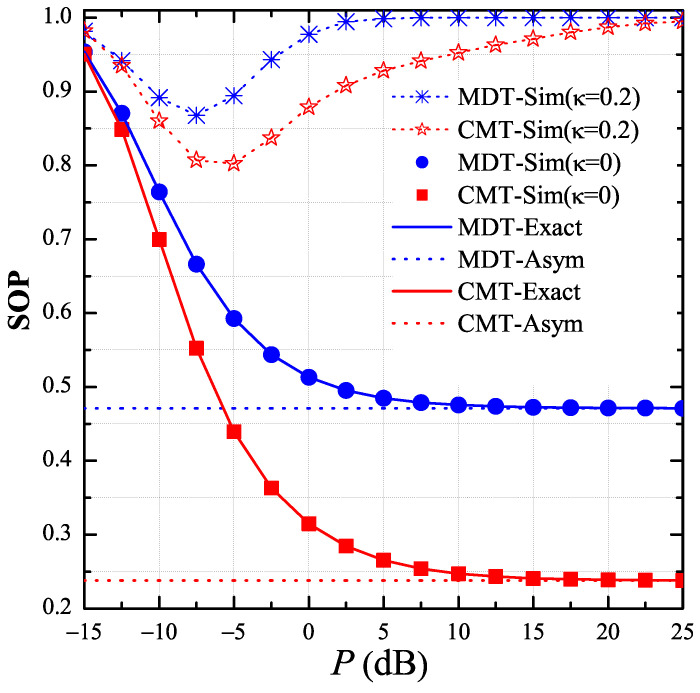
End-to-end secrecy outage probability (SOP) as function of *P* in dB when $P\in \left[-15\;\mathrm{dB},\;25\;\mathrm{dB}\right]$, μ=0.5, M=4, $R_{S}=1$, $\kappa \in \left\{0,\;0.2\right\}$, $\left(x_{\mathrm{PU}},y_{\mathrm{PU}}\right)=\left(-0.5,\;-1\right)$, $\left(x_{E},y_{E}\right)=\left(0.5,\;0.5\right)$ and $\phi_{D}=\phi_{P}=\phi_{E}=1$.

In Figure 3, we present the end-to-end SOP of the MDT and CMT protocols as a function of the transmit SNR $\left(P=P_{\mathrm{th}}/\sigma_{0}^{2}\right)$ in dB when all of the channel estimations are perfect, i.e., $\phi_{D}=\phi_{P}=\phi_{E}=1$. As we can see, the proposed protocol (CMT) outperformed the MDT protocol for all the *P* values because the destination and the intermediate relays in the CMT protocol could obtain higher diversity gain as compared with those in the MDT protocol. As a result, the proposed protocol enhanced the channel capacity of the data links, which hence provided better secrecy performance. In addition, it was observed that, when the transceiver hardware was perfect $\left(\kappa =0\right)$, the secrecy performance of both protocols converged to the asymptotic results, which were independent of the *P* values. However, as κ=0.2, the values of SOP reached 1 at high region, which validated the statement in Section 3. Moreover, there existed a value of *P* at which the value of SOP was lowest. As shown in this figure, the optimal transmit SNRs in the CMT and MDT protocols were −5 dB and −7.5 dB, respectively. Finally, it is worth noting that the simulation results (Sim) match very well with the theoretical results (Exact), and, at high *P* regimes, the simulation results nicely converge to the asymptotic ones (Asym). These validate the correction of our derivations expressed in Section 3.

**Figure 4:**
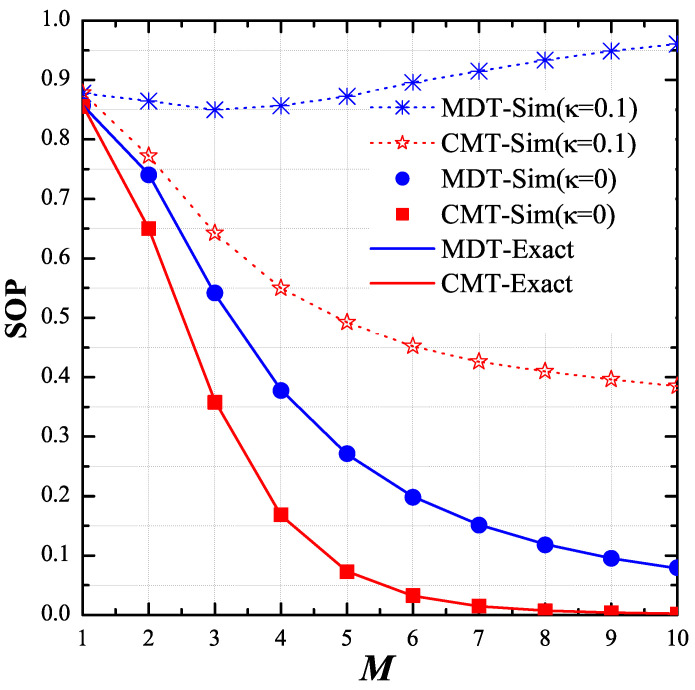
End-to-end secrecy outage probability (SOP) as function of *M* when P=5dB, μ=1, $M\in \left[1,\;10\right]$, $R_{S}=0.5$, $\kappa \in \left\{0,\;0.1\right\}$, $\left(x_{\mathrm{PU}},y_{\mathrm{PU}}\right)=\left(-0.5,\;-0.5\right)$, $\left(x_{E},y_{E}\right)=\left(0.5,\;0.5\right)$ and $\phi_{D}=\phi_{P}=\phi_{E}=1$.

As shown in Figure 4, we changed the number of hops (*M*) and observed the variant of the end-to-end SOP. We assigned the values of *P*, μ, $R_{S}$, $x_{\mathrm{PU}}$, $y_{\mathrm{PU}}$, $x_{E}$, and $y_{E}$ as 5 dB, 1, 0.5, −0.5, −0.5, 0.5 and 0.5, respectively. As observed, with the perfect transceiver, the secrecy performance of the MDT and CMT protocols was better when the number of hops increased. For the CMT protocol, this result is still true with the presence of the hardware imperfection (κ=0.1), while the performance of the MDT protocol severely degraded with higher number of hops. Again, the results in this figure validate the theoretical results provided in the previous section.

**Figure 5:**
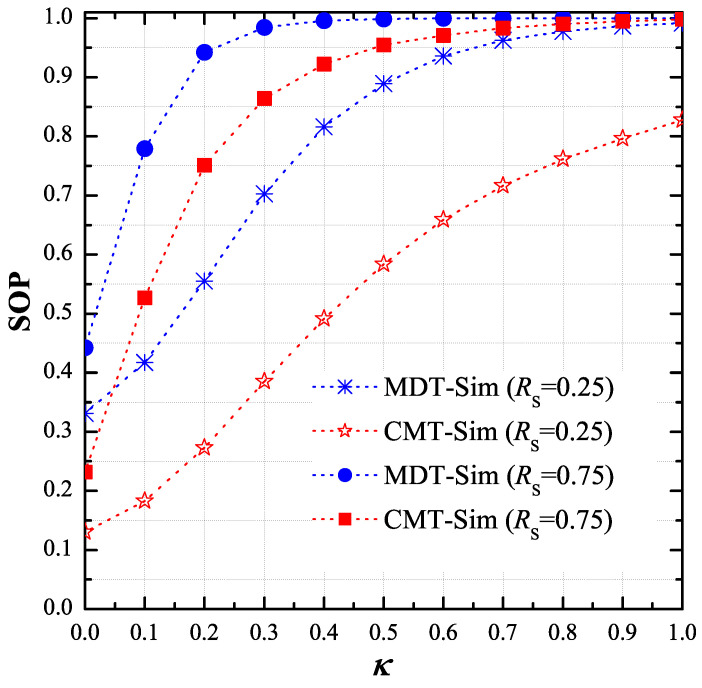
End-to-end secrecy outage probability (SOP) as function of κ when P=0dB, μ=1, M=4, $R_{S}\in \left\{0.25,\;0.75\right\}$, $\kappa \in \left[0,\;1\right]$, $\left(x_{\mathrm{PU}},y_{\mathrm{PU}}\right)=\left(-0.5,\;-1\right)$, $\left(x_{E},y_{E}\right)=\left(0.5,\;0.5\right)$ and $\phi_{D}=\phi_{P}=\phi_{E}=1$.

Figure 5 presents the impact of the hardware impairment level $\left(\kappa \right)$ on the secrecy performance of the CMT and MDT protocols when P=0dB, μ=1,M=4,$x_{\mathrm{PU}}=-0.5,$$y_{\mathrm{PU}}=-1,$$x_{E}=0.5$ and $y_{E}=0.5$. Similarly, the proposed scheme obtained better performance, as compared the MDT scheme. It is also seen in Figure 5 that the SOP values rapidly increase as the κ value increases. In addition, the performance of the considered methods significantly enhanced with lower value of the target rate $R_{S}$.

**Figure 6:**
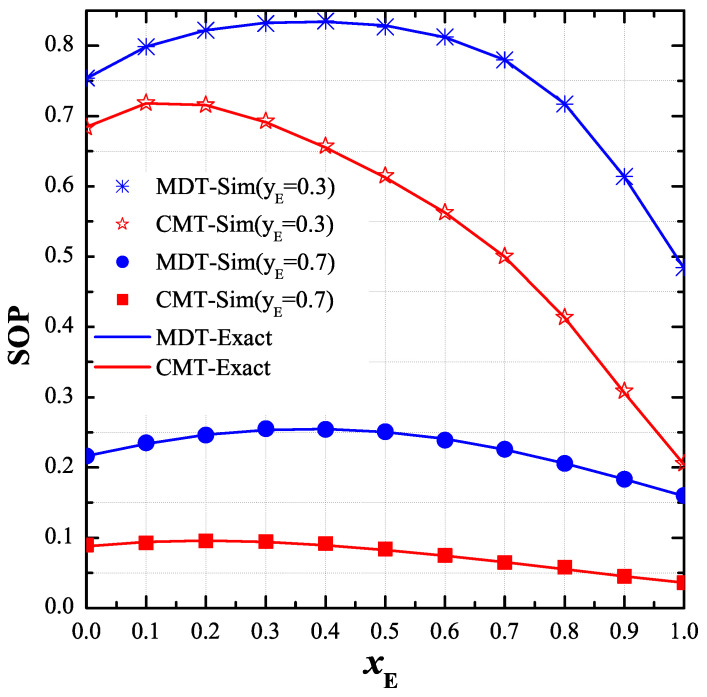
End-to-end secrecy outage probability (SOP) as function of $x_{E}$ when P=10dB, μ=1, M=4, $R_{S}=1$, κ=0, $\left(x_{\mathrm{PU}},y_{\mathrm{PU}}\right)=\left(-0.5,\;-1\right)$, $y_{E}\in \left\{0.3,\;0.7\right\}$ and $\phi_{D}=\phi_{P}=\phi_{E}=1$.

As shown in Figure 6, we studied the effect of the positions of the eavesdropper on the end-to-end SOP. In particular, we fixed the value of $y_{E}$ while changing $x_{E}$ from 0 to 1. The remaining parameters were set as: P=10dB, μ=1, M=4, $R_{S}=1$, κ=0, $x_{\mathrm{PU}}=-0.5$ and $y_{\mathrm{PU}}=-0.1$. It can be seen that the end-to-end SOP of the CMT protocol mostly decreased with the increasing of $x_{E}$, while that of the MDT increased at small $x_{E}$ value and decreased at high $x_{E}$ region. We can see in this figure that the performance of the MDT protocol was worst when $x_{E}$ was about 0.4.

## 5. Conclusions

In this paper, we propose the cooperative multi-hop transmission protocol (CMT) in the UCR networks with the presence of an eavesdropper. Because the proposed scheme uses cooperative multi-hop transmission, it significantly outperforms the conventional multi-hop direct transmission protocol (MDT), in terms of the end-to-end secrecy outage probability (SOP). The interesting results obtained in this paper can be listed as follows:

- The secrecy performance of the proposed protocol was much better than that of the MDT protocol when the CSI estimations of the data, interference and eavesdropping links were perfect. Otherwise, the SOP performance significantly degraded due to the incorrect relay selection.
- When the transceiver hardware of the nodes was imperfect, the secrecy performance severely degraded. In particular, the value of the end-to-end SOP rapidly increased with higher transmit signal-to-noise ratio (SNR) and with higher impairment level.
- In the presence of the hardware noises, there existed an optimal value of the transmit SNR, at which the secrecy performance of the CMT and DMT schemes was best.
- The performance of the proposed protocol was better when the number of hops was higher.
- When the hardware impairments were relaxed, we derived exact and asymptotic expressions of the end-to-end SOP for the CMT and MDT protocols. We then performed computer simulations to verify the derived expressions.
